# Remote Work Support Needs of Employees with Autism Spectrum Disorder in Poland: Perspectives of Individuals with Autism and Their Coworkers

**DOI:** 10.3390/ijerph191710982

**Published:** 2022-09-02

**Authors:** Michał T. Tomczak, Elias Mpofu, Nathan Hutson

**Affiliations:** 1 Faculty of Management and Economics, Digital Technologies Center, Gdańsk University of Technology, 80-233 Gdańsk, Poland; 2 Department of Rehabilitation and Health Services, University of North Texas, Denton, TX 76203, USA; 3 School of Health Sciences, University of Sydney, Lidcombe, NSW 2141, Australia; 4 Educational Psychology, University of the Witwatersrand, Johannesburg 2000, South Africa; 5 Department of Public Administration, College of Health and Public Service, University of North Texas, Denton, TX 76203, USA

**Keywords:** autism spectrum disorder, neurodiversity, remote work, hybrid work, well-being

## Abstract

Background and Aims: With remote work becoming more common across industries, employees with autism may experience different work support needs from neurotypical peers. However, the specific remote work needs of this group of employees are underexplored in the literature. We aim to propose ways to assess workplace digital adaptation needs for individuals with autism and a framework for communicating these needs to employers. Methods: This qualitative study included interviews with 13 Polish business professionals, including coworkers and/or supervisors of employees with autism (*n* = 9) and female employees with autism (*n* = 4), about their remote work support needs. Participants responded to semi-structured interview questions identifying advantages and risk factors associated with remote work for this specific group of employees. Results: Participants reported advantages of remote work, such as limiting sensory overload and intensive interpersonal contacts, indirect interpersonal communications, flexible work hours, and eliminating the need to travel to work. Participants also reported challenges of remote work, such as reducing wanted or helpful social contacts, engaging in direct electronic communications, limiting opportunities to learn from other employees, and managing work–life balance. Conclusion: These findings suggest a need for an autism-inclusive digitalized remote work design customized to the unique needs of employees on the autism spectrum. Business managers would be key partners in the design of autism-inclusive digitalized remote work systems. Additional research is needed with larger and more diverse samples of employees with autism.

## 1. Introduction

Remote working is fast becoming normalized and broadly accepted across industries. Remote work is when work that would have been traditionally performed in an in-person setting is digitalized for implementation away from the office [1]. It includes telework or telecommuting and minimal physical interaction with other coworkers during the course of the workday [2,3]. The benefits of in-person workforce participation to social functioning over time are well documented and include improved social relationships, community integration, improved sense of self-worth, and general quality of life [4,5,6]. However, in the increasingly digitalized work world [7], the strategies for integrating individuals with autism within remote employment settings are less certain. Remote working is not a one-size-fits-all solution to the needs of employees, nor is it clear how this distance work environment meets the work participation needs of employees with autism.

### 1.1. Autism and Work Participation

People with Autism Spectrum Disorder (ASD) present with a wide range of difficulties in interpersonal communication, social reciprocity, and sensory sensitivity [8]. These atypical social and communication differences may complicate their interactions with neurotypical peers [9,10,11], risking employment participation vulnerabilities in this population, which has an unemployment rate four times higher than that of the general population [12]. In many ways, workers with autism have a reason to embrace the concept of remote work, as it creates the opportunity to avoid uncomfortable informal social interactions that would otherwise interfere with job productivity [13]. On the one hand, workers with autism tend to benefit less from tacit social knowledge transfer than neurotypical colleagues [14]. On the other hand, the lack of informal social interactions may lead to the breakdown of learned social competencies, which may eventually inhibit these individuals’ ability to progress in their chosen profession and their overall social functioning beyond the office setting.

Of the studies that examined remote working experiences with neurodiversity (see [15,16]), most did not focus explicitly on employees with autism but included related conditions. The major exception is a study by Goldfarb et al. [17], who explored the remote digital work experiences of employees with autism associated with the digital transition in the context of the COVID-19 pandemic. We aimed to address the gap in research on the remote work support needs of Polish employees with autism from their own perspective and those of business professionals from the same country. 

### 1.2. Polish Autism-Inclusive Work Practices

Autism is under-reported in the Polish setting at 3.4 cases per 10,000 individuals. This estimate is vastly lower compared to 200 cases per 10,000 individuals in equivalent surveys in the United States [18]. The under-reporting is likely similar in other former Eastern Block states [19]. As Poland’s medical and psychological care system becomes more integrated with that of Western Europe, the reported autism prevalence may increase. 

Middle-aged to older adults with autism in the workforce are likely to be undiagnosed since diagnostic services tend to serve children and younger adults [18]. However, the legacy of underdiagnosis continues to result in a lower awareness of the autistic condition in general society, which will inhibit effective interventions, especially when these interventions are the responsibility not of medical professionals but of business professionals who have no formal training and little understanding of the condition. Regardless of whether they have received a formal diagnosis, neurodiverse adults in Poland are entering the workforce at a time when the norms surrounding work culture are in a state of flux. Specifically, the changing nature of remote work presents both opportunities and threats for neurodiverse workers. Polish employees with autism and business managers have little guidance on work adaptations for neurodiversity, let alone for remote working, which is becoming the new normal globally post-pandemic.

Remote work environments vary greatly across industries but are generally associated with the following work outcome goals: (1) less work absenteeism and a consequent increase in labor productivity [20]; (2) lowering the “tyranny of distance” and allowing employers to draw on labor from those who live in rural areas or “exurbs” [21]; and (3) improving employee perception of the company’s concern with employee welfare [22]. However, with the remote work culture for all employees still in a state of change, the norms that apply to employees with autism remain unclear. An advantage to businesses with remote work using digital tools is that flexi-work for all employees is associated with higher productivity, which is associated with fewer work disruptions. 

### 1.3. The Present Study

In Poland, the discussion of the work needs of individuals with autism is in its nascent stages and is only now gaining attention within the business community. The current study aimed to explore the evidence for the remote work support needs of employees with autism from the perspective of business professionals, including employees with autism and their colleagues and managers. Our specific research questions were:

1. What are the advantages of digitally supported remote work as perceived by workers on the autism spectrum and their coworkers?
2. What are the long-term challenges of digitally supported remote work as understood by employees on the autism spectrum and their coworkers?

The findings may provide evidence for autism-inclusive remote work arrangements that would also enhance business organization productivity. 

## 2. Methods

### 2.1. Research Setting and Design

We recruited a purposive sample of 13 participants utilizing snowballing (business professionals, including coworkers and supervisors of individuals with autism: *n* = 9, and employees with autism: *n* = 4). To be included in the study, participants with autism or those who worked with a coworker or supervisee with autism for at least 6 months self-reported their experience with remote work. The characteristics of the research participants are presented in Table 1. 

### 2.2. Data Collection

The research participants completed semi-structured interviews on their opinions on the remote work support needs of employees with autism, identifying any associated advantages and disadvantages of both forms of work. In addition, participants were asked to propose recommendations on how to improve the remote work experiences of employees with autism. Specifically, participants responded to the following basic framing of work participation aspects:

1. The respondent’s role in their organization.
2. Their attitudes about remote work, including the advantages and associated challenges from the perspective of a person with autism.
3. Their recommendations on how to improve the remote work experiences of individuals with autism.
4. Other information they may have that would contribute to the study aims.

Interviews lasted 18 min on average, and the average transcript length was 3 pages per interview. An additional advantage of the qualitative method for this study was the ability to establish trust with interviewees and to build relationships that engendered a deeper understanding of the research topic [23]. A semi-structured interview format was used [24].

### 2.3. Procedure

The interviews were conducted remotely by the first-listed author in late 2021 and early 2022 with the use of MS Teams software, in compliance with the ethical guidelines and procedures of the Gdańsk University of Technology (No. 303/2011). Participants individually consented to the study. Before each interview, participants received an invitation letter detailing the aims and procedure of the research study, asking them to take part in the research, ensuring confidentiality, and informing them of their right to withdraw at any moment. 

### 2.4. Data Analysis

We analyzed the interview transcripts utilizing the template analysis procedure [25,26]. Template analysis of the data involved the following steps: 1. becoming familiar with the accounts to be analyzed; 2. carrying out preliminary coding of the data; 3. organizing the emerging themes into meaningful clusters; 4. defining an initial coding template; 5. applying the initial template to further data and modifying them as necessary; and 6. finalizing the template to apply it to the full dataset. 

The initial analysis of the research data was conducted by the first author, who created the codes. The second- and third-listed authors were involved in cross-checking emerging themes [27], providing inter-coder reliability, and developing the final coding template [28]. To better interpret the themes, the research team consulted with employment support specialists at the University of North Texas’s ENGAGE Center for Neurodiversity and Workplace Inclusion and Sustainable Employment Center (UNT WISE), which aided in contextualizing the evidence.

## 3. Results

Several themes emerged from the analysis (see Table 2). Participants reported the following advantages of remote work: limiting sensory overload, limiting intensive interpersonal contacts, engaging in indirect electronic communications, availability of flexible work hours, and eliminating the need to travel to work. Participants reported the following challenges of remote work: reducing wanted or helpful social contacts, engaging in direct electronic communication, limiting opportunities to learn from other employees, and managing work–life balance. We provide evidence for each of these themes below.

### 3.1. Advantages of Remote Work

#### 3.1.1. Limiting Sensory Overload

All of the research participants stated that employees with autism perceive a remote work advantage associated with overcoming the limitations related to sensory sensitivity (auditory, sound, olfactory, or visual), which is usually not possible in a stationary office environment. For example, one of the respondents made the following observation:

*“Remote work offers the possibility of limiting sensory overload. Because, e.g., even if there is a chillroom in the office, it is sometimes used as a chat room, not in accordance with the intended use”.*

(R11)

Due to unique sensory sensitivities while working from home, some individuals with autism may find it easier to adapt the home environment to fit the best sensory needs and preferences:

*“There is a possibility of sensory adaptation to our needs, because each person on the spectrum has different needs in this area. At home, we are able to adjust the conditions individually.”*

(R11)

*“In the case of olfactory hypersensitivity, the workplace should be at a distance from the kitchen or break room, or the solution may also be a possibility of self-preparation and consumption of a meal alone. In the case of sound hypersensitivity, the workstation should be away from corridors or staircases, where fewer distractors occur. Enabling individualization as needed is also highly recommended, as some individuals prefer dark or light rooms, others prefer high or low sounds. The work environment should include neutral or pastel colors of office rooms to not arouse interest in the space and avoid motley and asymmetrical patterns. However, it is not possible to arrange an office to suit everyone, and at home everyone can do it individually.”*

(R7)

#### 3.1.2. Limiting Intensive Interpersonal Contacts

In this context, it is also worth mentioning that working remotely supports the limitation of intense and uncomfortable interpersonal contact, mainly within large groups, which can also be perceived as highly burdensome and stress-inducing to people with autism. This was indicated by ten research participants, including all four interviewees on the autism spectrum.

*“They (employees on the autism spectrum) praise remote work very much, mainly due to the fact that in the case of remote work there is no small talk, big meetings, and activities that last a very long time”.*

(R6)

*“The obligation to participate in small talk, conversation, etc. disappears. This is a big relief. The less the mental strain, less stress, especially with a new client. It is much easier when online.”*

(R12)

#### 3.1.3. Engaging in Indirect Electronic Communications

Two of the research participants also mentioned that remote working favors using impersonal and electronically mediated communication (in the form of e-mails, chats, instant messaging, chatbots, etc.), which could also significantly improve communication for people for whom face-to-to-face contact is a considerable challenge [29,30]. Not only do electronic messages tend to “get to the point” with fewer potentially distracting phrases, but they can also include real-time feedback on the responses drafted by the individual with autism [31]. For example, e-mail programs include content analysis that notifies the writer when their tone may sound rude to the recipient party. These types of internal governors on the tone of written speech can give the worker on the autism spectrum greater confidence in their responses and can therefore empower the individual in the same way that specialized spellcheck software can aid those with dyslexia:

*“The nature of electronic communication, which is easier to control, does not require an immediate verbal response, prompts to be more careful when expressing thoughts and feelings, is an advantage making it more suitable for autistic employees.”*

(R7)

#### 3.1.4. Availability of Flexible Work Hours

As indicated by another two respondents, there are business organizational advantages in implementing greater flexibility in work scheduling for people with autism, including flexible working hours and the use of a task-based approach instead of hourly measurements of working time and productivity. It should be noted, however, that many individuals with autism have a comorbid condition of attention deficit disorder [30]. Thus, complete flexibility of work scheduling may sometimes be problematic:

*“Working hours, despite the fact that they are, somehow framed there but they are not so rigid. My point is, you can take a break when you need it. And to make up for something even after working hours. It is important to complete the task. Therefore, there is accounting for the task, not for minutes at work or minutes in front of the computer. And these people are doing great with it.”*

(R6)

#### 3.1.5. Eliminating the Need to Travel to Work

According to the statements of two interviewees (both with autism), avoiding physical travel is another large benefit for those on the spectrum. While specific data for Poland are not yet available, generally, adults with autism are much less likely to hold a valid driver’s license than the general population and are thus overly reliant on public transport [32]. Even when the individual has a viable way to access the office, eliminating the necessity of daily travel between home and the workplace translates into saving time and money and additionally reduces the impact of sensory stimuli [17]. While the stress of a daily commute is not unique to individuals with autism, any factor that can allow the individual to start their day in a lower-stress state can aid in the individual’s ability to “make it through the day” and should be welcomed—as indicated by the following respondent statements.

*“You don’t have to travel to work, especially by public transport. It was tiring due to crowds, noise, other stimuli and could be a stressor itself.”*

(R11)

*“No need to travel, stay in large groups of people, no feeling of being watched by other people”.*

(R13)

### 3.2. Challenges of Remote Work

#### 3.2.1. Reducing Wanted or Helpful Social Contacts

Eight respondents, including all four interviewees with autism, perceived a need for social contact support to prevent unwanted social isolation. For instance, one respondent stated:

*“It’s not about limiting any kind of contact. Because pragmatic contact is appreciated by many people. So in this sense, it is about limiting contact with some group of associates, not necessarily all of them.”*

(R7)

Moreover, limiting social contact may result in fewer opportunities to offer developmental challenges related to crossing the comfort zone that may be uncomfortable in the short term but result in long-term skill acquisition. The lack of these challenges may negatively impact social integration over the long term.

*“I am a supporter of giving a person with autism adequate social challenges, which is also a development challenge. So, I am not a supporter of the situation to enclose these people with maximum comfort and everything, because the whole thing will tend to entropy. People need development challenges adequate for them, at the optimal level of stress. However, (some) stress. And now, if we isolate anyone, anyone with a super comfortable, ideal life on all sides, it does not bode well for us.”*

(R9)

#### 3.2.2. Engaging in Direct Electronic Communications

Similarly, seven interviewed business professionals perceived remote working as a challenge for efficiently structuring work activities, including effective communication using a virtual forum, such as a zoom call (see also [29]). One of the business professionals made the following observation:

*“Some autistic people have trouble turning on their webcam. They may even prefer face-to-face contact, rather than meeting on a webcam. It depends on the individual”.*

(R12)

Some people with autism may struggle with difficulties in executive function and task ordering with remote work. In other words, while the individual on the autism spectrum may prefer a remote setting with less strict deadlines, over time, this could impede work progress and the ability to meet deadlines.

*“Some people on the autism spectrum face a problem with answering the phone, a problem with reporting some issues that arise, and with asking for help when it happens”*.

(R7)

*“There was a decline in online meetings, because no one pushed them to do so. Only a few people were performing these tasks. Half of the group, even a little more than half of the group, did not.”*

(R3)

#### 3.2.3. Limiting Opportunities to Learn from Other Employees

During the interviews, three research participants shared the opinion that remote work can also lead to challenges in conducting effective training, acquiring knowledge, and learning [17], mainly in the onboarding phase but also as part of the job retention stage. Orientation into a new job function is always challenging, but without in-person feedback in the early stages of employment, incorrect work habits can become ingrained.

*“Providing opportunities to communicate and learn from other employees became a challenge for a few of people that I’ve worked with. Because they’ve been working remotely, they haven’t been able to learn from more experienced people in the workplace because they are not able to shadow them in their tasks.”*

(R2)

This is a problem in general with remote workers, but individuals with autism may need more frequent guidance and may take longer to acclimate to work culture [33]. Moreover, every worker, regardless of their disability status, will respond differently to different forms of correction by a supervisor. Whereas an individual with autism may have difficulty in reading the body language of a supervisor [15], a supervisor may need training on providing feedback in a constructive way to employees with autism without causing unwanted anxiety [29]. One respondent pointed to the importance of training and feedback.

*“Remote work is much worse in the education and training sphere. And this educational space is also those places where there are no clearly formulated expectations on the other side.”*

(R6)

#### 3.2.4. Managing Work–Life Balance

In accordance with the statement of one research participant, remote work may also come at a cost to the work–life balance of employees with autism. This concern was also raised by an outside job coach interviewed as background for the study [29]. As mentioned before, a significant percentage of employees with autism also have challenges with executive functioning and/or a comorbid ADHD diagnosis [30]. Both of these conditions can inhibit the ability of the employee to prioritize tasks. 

In a situation where work time is less structured, it may be more difficult to define a clear division into work and leisure. This can lead to a situation where an employee spends an inordinate amount of time working to the long-term detriment of his/her mental health. In addition, when an employee is not physically seen, warning signs that they are working excessive hours are more difficult for an employer to discern.

*“For some people with autism, this is a problem. They felt the need to change the work environment and the need for the work-home division.”*

(R13)

All themes and subthemes identified above, along with examples of quotes from the statements of the respondents, are summarized in Table 2. 

### 3.3. Suggestions for Improvement

#### 3.3.1. Reducing Helpful or Wanted Social Contacts

Six research participants proposed hybrid work arrangements as a solution to remote work challenges in which they can engage in partial remote work participation, retaining the advantages of both remote and in-person work. For instance, one respondent said:

*“There have been a few individuals that I’ve been working with at the moment who are working from home. Some absolutely are thriving and loving it. And its half that are actually not loving it because they are not around people to have the social interaction and things like that. So I think it’s again dependent on the individual and I guess their experience.”*

(R2)

This speaks to the fact that people with autism have unique socialization needs that must be recognized and respected in work settings. 

#### 3.3.2. Engaging in Direct Electronic Communications

For two employees with autism, hybrid solutions that would work best are those that included tools for efficient and clear communication:

*“e.g., the benefits of chatbots are that they are based on rules, precise, and encourage messages without accents and ambiguities, which are difficult to comprehend for employees with ASD.”*

(R7)

An additional strategy would be allowing enough time for asking questions, complementing verbal instructions in written form, and providing summaries after meetings. As one of the interviewees emphasized:

*“Having more time to ask questions and setting minutes after meetings is really helpful.”*

(R2)

Finally, one employee with autism noted that she would appreciate regular, constructive, and honest feedback to avoid misunderstandings. As mentioned previously, attaining a good balance between feedback that is constructive without being excessively harsh will almost always be more challenging in a virtual setting, where tacit body signals are missing.

*“It is a matter of precision, clear expectations. What am I supposed to do? And judging me for what I did. This remittance is successful when there is a partner on the other side, someone who is able to carry out this remotely.”*

(R7)

These observations highlight the need for various social and communication supports for employees with autism to improve work engagement in positive ways. 

#### 3.3.3. Limiting Opportunities to Learn from Other Employees

Two business professionals considered technology-based flexible forms of contact with the manager and team members to be important. One respondent said:

*“It is worth creating an application so that a person with autism can contact a coach, e.g., once a week. In practice, constant contact with the coach is impossible, because a good coach is very busy. However, I would like it to be an application where you can ask a question and get an answer. Even as a kind of communicator.”*

(R9)

From this evidence, inclusive communication with employees across the neurodiversity spectrum may be enhanced with the use of tools that a job coach and employee would use in tandem to achieve work goals. This would require business managers to recognize that providing inclusive communication support is part of good business practice. 

#### 3.3.4. Managing Work–Life Balance

In this case, the role of the manager or job coach is very important, consisting of support in defining a clear division into work and leisure, making it easier to prioritize tasks, avoid excessive working hours, and help workers self-advocate for these goals. In the case of remote work, an application supporting task prioritization and time management to better control working time would be advantageous. One of the respondents stated:

*“To overcome the limited contact with the supervisor, the application could be a good idea for the team. Its task would be to support in setting priorities, order of tasks monitoring and their execution. It would be a great help.”*

(R3)

This evidence illustrates the new-normal future of remote working, in which the use of applications will be embedded in most work routines across the neurodiversity spectrum. 

## 4. Discussion

The advantages of remote working include work opportunities, flexible commuting, higher work flexibility, and work satisfaction. The findings of the study are in line with the results of previous studies that examined remote work from the perspective of the general population [34,35,36]. Furthermore, remote working was preferred by others among the neurodivergent population, including individuals with ADHD, dyslexia, dyspraxia, and autism (see [15,16,17]). Our research results provide evidence on the preferences and priorities of people with autism, each of whom is unique in their sensitivity to sensory overload, and for whom indirect electronic communications can be highly beneficial, allowing the limitation of intensive interpersonal contacts.

Similarly, while social distancing [37,38] in remote work settings may be beneficial to employees on the spectrum who seek less intensive interpersonal communication, they may also inadvertently limit opportunities to learn from other coworkers [17]. While autism is a social and communication disorder [9,11,15], people with autism have unique socialization needs to be “accepted or appreciated as an autistic person, with autism positively recognized and accepted by others and the self as an integral part of that individual” [39] (p. 424). Our findings underscore the fact that unique sociality needs with autism should be understood alongside strengths. Remote work supports for those with autism need to highlight the fact that sociality with autism requires an awareness by business managers to customize work learning experiences to social accessibility of communication. 

Our findings suggest a challenge to employees with autism for achieving a better work–non-work balance by navigating their work tasks while not sacrificing their other necessary (non-work life) activities (see also [16,40,41,42]). Goldfarb et al. [17] noted that an increase in work–life balance is presented as an advantage of remote working due to flexible work scheduling. While possible, achieving this outcome may be more difficult for the autistic population due to difficulty defining and limiting work hours and blurred boundaries between work and home.

### 4.1. Implications for Research and Practice

Although remote working may be advantageous to work scheduling autonomy, some people may experience difficulties structuring their work activities at home in a way that does not take up excessive time. For managers, this presents a challenge, as optimal work schedules will vary by individual—each of whom will have their own definition of the optimal work–life balance. One strategy for managers is to monitor sudden changes in online engagement over time. For example, an employee who used to log off of their station by the end of the workday but is now staying logged in until late at night can be seen as a warning sign of a breakdown in executive function. These types of work pattern changes could be regarded as a flag for the manager to check in on the employee. 

Often, the benefits of limiting unwanted interpersonal contact will aid work efficacy [43]. Nevertheless, while reducing unwanted stimuli, for some people, it also carries a risk of workplace social exclusion with implications for career trajectory. In general, the employee with autism may be less likely to seek out discretionary workplace social interactions. Thus, it is incumbent on the manager to be creative in designing semi-structured forums for the remote employee to engage with coworkers. Relatedly, the manager may need to take extra precautions to ensure that online bullying, harassment, and/or exploitation by coworkers does not occur, as these practices may be more difficult to detect in a virtual setting [44].

Many of the strategies that have been previously proposed for workplace adaptations are transferable, with minor adjustments, to the remote setting. In some contexts, providing a mentor may be an effective strategy, particularly when direct monitoring by a supervisor is not possible [44]. Supervision and assistance by coworkers or managerial staff can be achieved using computer software or mobile devices [7,43,45]. If properly executed, this can be a benefit to overall work efficiency, assuming that supervisors are properly trained in the needs of employees on the autism spectrum with a shift to virtual employment. 

Furthermore, general suggestions for managers that can be considered include: assessing the needs of employees with autism to enhance the advantages of remote work; offering hybrid work options and opportunities for social interaction; encouraging the use of chats during online meetings; and having technology-based forms of contact with managers and team members.

### 4.2. Study Strengths and Limitations

This research has significant potential in the area of supporting people with autism in adjusting to the evolving post-pandemic work culture that, despite its challenges, may be a net benefit for neurodivergent employees. Given the diversity of presentation, semi-structured interviews proved to be a productive tool for elucidating a range of potential strategies. This is the first study focused directly on the remote work support needs of employees with autism in Poland, where the condition remains underdiagnosed and poorly understood amongst working adults. The study provides preliminary evidence on evidence-based practices for the work well-being and productivity of employees on the autism spectrum. This study also has some limitations. First, due to the small research sample from only one country and the fact that, among research participants with autism, diagnoses were only in women completing interviews, additional studies may be needed to check on the dependability of our observations. In addition, the respondents to this study diagnosed with autism were fully verbal, high-functioning self-advocates. The findings may be different for other employees with less verbal ability. A related limitation is that the study reported on diagnosed and self-identified people with autism, and it may have missed others who are undiagnosed. Future studies should seek to interview a diversity of participants on the spectrum and with higher enrollment of those with autism than was possible in this study. 

## 5. Conclusions

Today, remote work has become ubiquitous and is becoming the new normal across industries. The speed at which this transition occurred was unprecedented and was spurred by the unlikely convergence of advances in communication technologies and a pandemic. For employees with autism in Poland, the “new normal” of remote work may run the risk of leaving them behind in the absence of evidence on perceived advantages and challenges associated with remote work. Remote work cannot be seen as a panacea for neurotypical employees. The same conclusion holds for their neurodiverse colleagues. Nevertheless, with proper accommodations, such as recognizing the needs of employees, providing hybrid work options, enabling the use of chats during online meetings, and supporting technology-based forms of contact, the new normal of remote work following the COVID crisis has the potential to become a net positive for the neurodiverse population in their quest for equitable treatment to neurotypical peers. To further these initial findings, a more systematic analysis of diverse samples is needed to compare our results to other types of employees and industries in Poland and other countries.

## Figures and Tables

**Table 1:** Detailed information on the research sample.

No	Gender	Position/Area of Activity	Career Stage	Type of Industry	Autism Diagnosis
1	M	Therapist working with adults with autism	Mid-career	Healthcare and SocialAssistance	No
2	F	Coworker of employee with autism	Early career	HR consulting	No
3	M	Coworker of employee with autism	Mid-career	Management consulting	No
4	M	Manager of employee with autism	Mid-career	Management consulting	No
5	M	Manager of employee with autism	Late career	HR consulting	No
6	F	Manager of employee with autism	Late career	Textile industry	No
7	F	Therapist working with adults with autism/Self-advocate	Mid-career	Healthcare and Social Assistance	Yes
8	F	Job trainer working with employees with autism	Mid-career	HR consulting	No
9	M	Job trainer working with employees with autism	Mid-career	Healthcare and Social Assistance	No
10	M	Manager of employee with autism	Mid-career	Printing services	No
11	F	Psychologist/self-advocate	Early career	Business training services	Yes
12	F	Neurodiversity and inclusion trainer/self-advocate	Early career	Business training services	Yes
13	F	Neurodiversity and inclusion trainer/self-advocate	Early career	Business training services	Yes

Source: Authors’ own research.

**Table 2:** Advantages and long-term challenges of remote work from the perspective of employees with autism.

Theme	Subtheme	Sample Quotation
Advantages of remote work	Limiting sensory overload	Working from home should generally be better. There are no such unexpected stimuli (R3)
		I don’t like when someone walks behind my back, so I don’t like the door behind me. There is no problem with this at home (R11)
	Limiting intensive interpersonal contacts	We do not have to contact other employees directly. An online meeting is more beneficial because there is no continuous, intensified and direct contact as in the office, where you are among people all the time, what is very burdensome and leads to overstimulation (R11)
	Engaging in indirect electronic communications	The possibility of contact through the communicator, with one person. Or that there is one designated person to contact, especially in such organizational cultures that have many bosses (R7)
	Availability of flexible work hours	Flexible working hours, according to your needs. We work during hours when we are most effective and not during rigid hours (R11)
	Eliminating the need to travel to work	Some of the difficulties related to work disappear when you change the mode of work to remote, e.g., sensory difficulties, or commuting. It’s convenient and also time saving (R12)
Challenges of remote work	Reducing wanted or helpful social contacts	People with autism are very fond of interacting with people, but only cool people (R6)
		I think it’s difficult to create that environment with any kind of contact, with social contact. It would not be advisable (R8)
	Engaging in direct electronic communication	The lack of stimulus from the supervisor (R3)
		There are also people who have such disturbed executive functions that working from home they have more trouble to mobilize to work (R7)
	Limiting opportunities to learn from other employees	It is more difficult to learn and acquire knowledge transferred remotely (R12)
	Managing work–life balance	There is no clear division between work and home, and maintaining work-life balance (R13)

Source: Authors’ own research.

## Data Availability

The dataset used and analyzed during the current study is available from the corresponding author upon request.
